# Long-term transplant outcomes after allogeneic hematopoietic transplant in pediatric patients with hematological malignancies are influenced by severe chronic graft vs. host disease and immune reconstitution

**DOI:** 10.3389/fped.2022.947531

**Published:** 2022-08-12

**Authors:** Blanca Molina, Marta González-Vicent, Ivan Lopez, Alba Pereto, Julia Ruiz, Manuel Ramirez, Miguel A. Díaz

**Affiliations:** Hematopoietic Stem Cell Transplantation Unit, Department of Pediatrics, Hospital Infantil Universitario “Niño Jesús”, Madrid, Spain

**Keywords:** chronic GvHD, immune reconstitution, children, landmark analysis, long-term follow-up, allogeneic HSCT

## Abstract

Long-term follow-up studies are crucial to ensure surveillance and intervention for late complications after allogeneic stem cell transplantation, but they are scarce on the pediatric population. This study aims to analyze risk factors for long-term transplant outcomes. We report a landmark analysis of 162 pediatric patients who underwent allogeneic transplantation between 1991 and 2016, and survived for at least 12 months after the transplant. With a median follow-up time of 10 years for the survivors, the probability of disease-free survival (DFS) and overall survival (OS) is 81 ± 3 and 88 ± 2%, respectively. Variables that influenced DFS in the univariate analysis were: disease phase (early phase 87 ± 3% vs. advanced phase 74 ± 5%; *p* = 0.04), acute graft vs. host disease (aGvHD; yes 73 ± 5% vs. no 87 ± 3%; *p* = 0.038), severe chronic GvHD (cGvHD; yes 41 ± 13% vs. no 85 ± 3%; *p* = 0.0001), and CD4+ lymphocytes 2 years after the transplant (above the median of 837/μl 98 ± 2% vs. below the median 82 ± 6%, *p* = 0.026). However, in the multivariate analysis, the only variable that influenced DFS was presence of severe chronic GvHD (yes vs. no, HR 6.25; 95% CI, 1.35–34.48; *p* = 0.02). Transplant strategies should aim to reduce the risk of severe cGvHD. Immune reconstitution surveillance may help clinicians to better deal with late transplant complications.

## Introduction

Nowadays, allogeneic hematopoietic stem cell transplantation (HSCT) is considered an accepted treatment for pediatric patients with high-risk hematological malignancies. Transplant failure mainly occurs in the first year after transplantation, and then it rapidly declines over time. Long-term follow-up is crucial for hematopoietic cell transplantation care to ensure surveillance and intervention for complications. Most long-term reports are focused on adults (1, 2), and data on children are limited (3, 4). Herein, we report a long-term follow-up study on risk factors that influence transplant outcomes of pediatric patients with hematologic malignancies that survived beyond 1 year after allogeneic transplant.

## Patients and methods

One hundred and sixty-two pediatric patients with hematological malignancies that underwent HSCT between 1991 and 2016 and survived for at least 12 months after the transplant were included in the study. There were 129 cases of first HSCT, 30 second cases of HSCT, and 3 cases of third HSCT. Acute lymphoblastic leukemia (ALL) was the cause of transplantation in 56% of the patients. Most patients (51%) were transplanted in second CR or more advanced disease defined as beyond second CR or active disease at time of transplantation. Related donors were preferred used (74%) with haploidentical donors in 30% of cases. Mobilized peripheral blood (82%) was the main hematopoietic stem cell source used. “*Ex vivo*” T-cell depletion techniques were used in 105 (65%) out of the 162 transplantation procedures. By the time of the study, 76 (47%) patients have had any grade of acute graft vs. host disease (aGvHD) (14 grade I, 34 grade II, 24 grade III, and 4 grade IV). At the time of study, 65 of the patients had neither aGvHD nor chronic GvHD (cGvHD), aGvHD was observed in 10, and 87(54%) had cGvHD (classical cGvHD in 65 and overlap syndrome in 22 patients). Their clinical severity was mild in 24 cases (26%), moderate in 45 (52%), and severe in 18 (21%). The median time from HSCT to onset of cGVHD was 4 months (range: 3–146 months), and the median duration of cGVHD at the time of analysis was 120 months (range: 2–295 months). Patients’ parents and/or their legal guardians gave written informed consent for transplant procedures, and the study was in accordance with the Declaration of Helsinki. The main patient, donor, and transplant characteristics are provided in Table 1.

**TABLE 1:** Patient, donor, and transplant characteristics.

Characteristic/variable	Numbers
Number of patients	162
**Clinical data**	
**Patient-related**	
Age at HCT, median (range), years	7 (1–20)
Gender: male/female	97/65
Lansky score at transplantation	90 (70–100)
**Disease-related**	
Diagnosis	
ALL	90 (56%)
AML	59 (36%)
CML	13 (8%)
**Disease status at transplant**	
1st CR	80 (50%)
2nd CR	46 (28%)
>2nd CR or active disease	36 (22%)
**Donor-related**	
Donor type	
MRD	72 (44%)
Haploidentical donor	48 (30%)
MUD	42 (26%)
**Graft-related**	
Graft source	
PBSC	133 (82%)
Bone marrow	16 (10%)
Umbilical cord blood	13 (8%)
Graft manipulation (*ex vivo* T-cell depletion)	
Yes	105 (66%9
No	57 (34%)
CD34+ cells (×10^6^/kg) infused	6.20 (0.19–68.5)
**Lab data**	
**Immune reconstitution 1-year post-transplant; median (range)**	
NK-cells/μl	168 (0–1,778)
CD3+ cells/μl	1,181 (99–9,487)
CD4+ cells/μl	483 (31–2,976)
CD8+ cells/μl	560 (24–8,150)
B-cells/μl	421 (0–5,708)
CD4/CD8 ratio	1.06 (0.05–6.03)
**Immune reconstitution 2-year post-transplant; median (range)**	
NK-cells/μl	152 (14–1,198)
CD3+ cells/μl	1,786 (279–6,151)
CD4+ cells/μl	837 (115–4,748)
CD8+ cells/μl	703 (73–3,291)
B-cells/μl	566 (0–4,523)
Median follow-up of survivors (range), years	10 years (2–27)

ALL, acute lymphoblastic leukemia; AML, acute myeloid leukemia; CML, chronic myeloid leukemia; CR, complete remission; PBSC, peripheral blood stem cell; MRD, matched related donor; MUD, matched unrelated donor.

### Conditioning regimen and pharmacological graft vs. host disease prophylaxis

Conditioning regimens were previously described in detail (5–8). They were TBI-based in of the 17 cases. One-hundred and forty-five of the patients were conditioned using a TBF regimen ± thymoglobulin. They consisted of intravenous fludarabine at 30 mg/m^2^/day for 5 days (days −6 to −2), intravenous busulfan administered once daily according to patient body weight for 3 days (days −5 to −3), intravenous thiotepa at 5 mg/kg/day for 2 days (days −3 to −2), and intravenous methylprednisolone at 5 mg/kg/day (days −6 to −2). Busulfan weight-based dosing was as follows: <9 kg: 1 mg/kg/dose; 9–16 kg: 1.2 mg/kg/dose; 16–23 kg: 1.1 mg/kg/dose; 23–34 kg: 0.95 mg/kg/dose; >34 kg: 0.8 mg/kg/dose (5). For patients who needed a second or third HSCT because of graft failure (6), the conditioning regimen consisted of fludarabine 40 mg/m^2^/day from days −5 to −3, thymoglobulin 2 mg/kg/day from days −5 to −3, and melphalan 120 mg/m^2^ on day −1. Cyclosporine with or without methotrexate was used as pharmacological GvHD prophylaxis from day −1 to engraftment, and it was tailored after the transplant while aGvHD was not present.

### Immune reconstitution analysis

Phenotyping of NK cells, T lymphocytes, T lymphocyte subsets, and B lymphocytes was performed on fresh samples of whole blood by multi-parametric flow cytometry as previously described (9). Data regarding immune reconstitution at the time of analysis are given on Table 1. Patients younger than 7 years had better CD3+ cell reconstitution, 2,202/μl (range: 1,053–6,151) and CD4+ cells, 1,044/μl (range: 115–4,748) than older ones: CD3+ cells 1,678/μl (range: 279–5,037) and CD4+ cells 680/μl (range: 196–2,030) (*p* < 0.01). More detailed data on immune reconstitution in patients with or without cGvHD are provided in Table 2.

**TABLE 2:** Immune reconstitution.

Variable	Chronic GvHD	No-chronic GvHD	*P*-value
Patients	*N* = 87	*N* = 65	
**1-year post-transplant**			
CD3+ cells/μl	1,150 (99–9,487)	1,263 (114–438)	n.s.
CD4+ cells/μl	412 (31–2,976)	575 (55–2,598)	0.002
CD8+ cells/μl	566 (33–8,150)	551 (24–2,696)	n.s.
NK cells/μl	170 (18–1,778)	166 (0–596)	n.s.
B cells/μl	364 (0–5,708)	523 (0–1,657)	**0.03**
**2-year post-transplant**			
CD3+ cells/μl	1,959 (279–5,037)	1,756 (932–6,151)	n.s.
CD4+ cells/μl	912 (115–4,308)	795 (353–4,748)	n.s
CD8+ cells/μl	733 (73–3,291)	617 (269–1,794)	n.s.
NK cells/μl	196 (16–1,198)	120 (14–551)	n.s.
B cells/μl	706 (0–4,523)	480 (91–1,916)	n.s

GvHD, graft vs. host disease.

Data are given as median and range. Bold values mean statistically significant.

### Study design, definitions, and statistical analysis

This is a retrospective study. The major study endpoints were disease-free survival (DFS), overall survival (OS), cumulative incidence of relapse (CIR), and non-relapse mortality (NRM) at several landmark times following transplantation.

Disease-free survival was defined as time from transplantation to relapse or death of any cause, whichever occurred first. aGvHD was graded according to standard criteria, whereas cGvHD was defined as mild, moderate, or severe according to NIH criteria (9, 10). We also analyzed the composite outcome cGvHD and relapse-free survival (CRFS), defined as the absence of cGvHD requiring systemic treatment, relapse, or death of any cause.

Disease-free survival was estimated using the Kaplan–Meier method. Cumulative incidence was used to estimate relapse and NRM. A multivariate analysis of survival was evaluated using the Cox proportional hazard regression model. A *p*-value < 0.05 was considered statistically significant. The statistical analyses were performed with the SPSS software (version 20.0; SPSS Inc., Chicago, IL, United States) and the R in Mac (version 3.3.1; Vienna, Austria).

## Results

**FIGURE 1:**
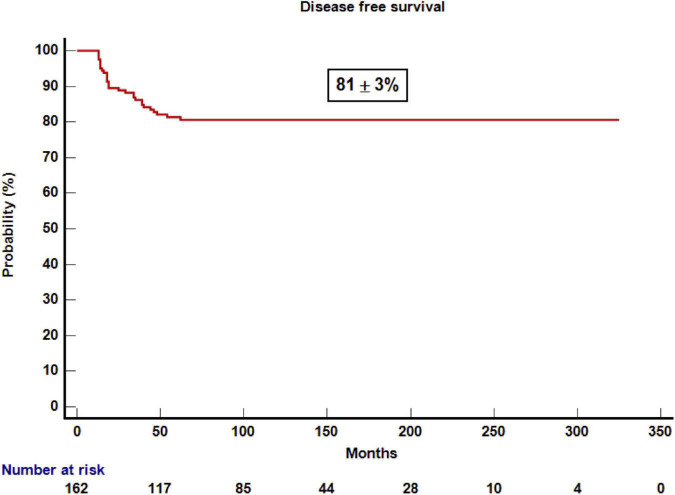
Kaplan–Meier estimation of overall survival after 1 year post-transplantation.

**FIGURE 2:**
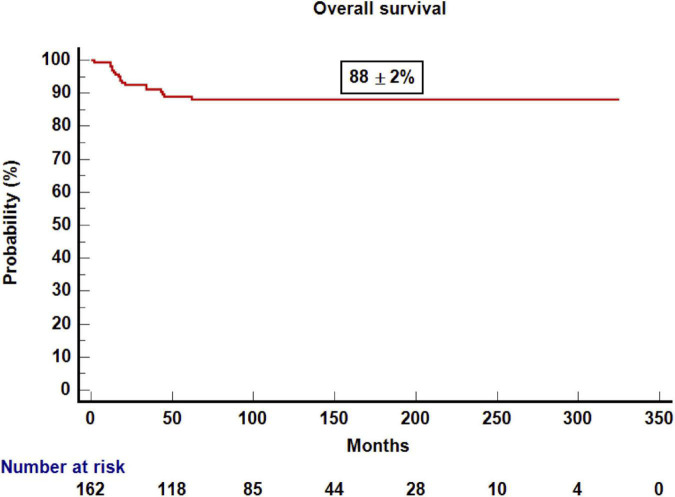
Kaplan–Meier estimation of disease-free survival after 1 year post-transplantation.

**FIGURE 3:**
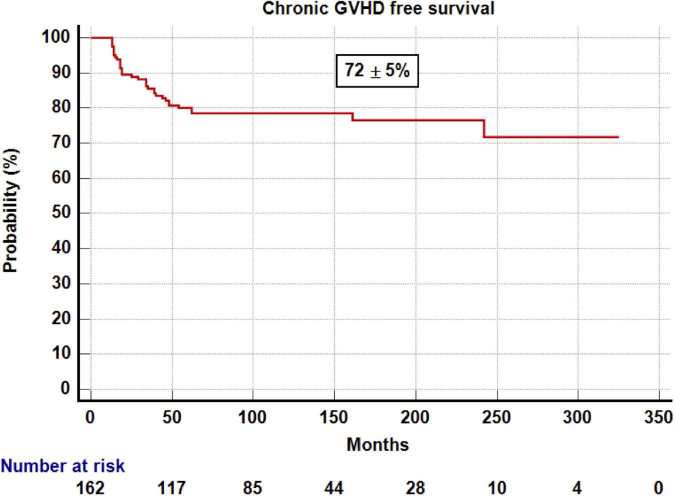
Kaplan–Meier estimation of CRFS after 1 year post-transplantation.

With a median follow-up for survivors of 10 years, the probability of DFS and OS was 81 ± 3 and 88 ± 2% (Figures 1, 2), respectively. The probability of CRFS was 72 ± 5% (Figure 3). At the time of writing, 143 of the patients were alive and disease-free. Nineteen of the patients died after 1 year. Infections were the most common cause of death (*n* = 7) (37%), followed by relapse (*n* = 6) (33%), GvHD (*n* = 3) (15%), and other causes (*n* = 3) (15%).

**FIGURE 4:**
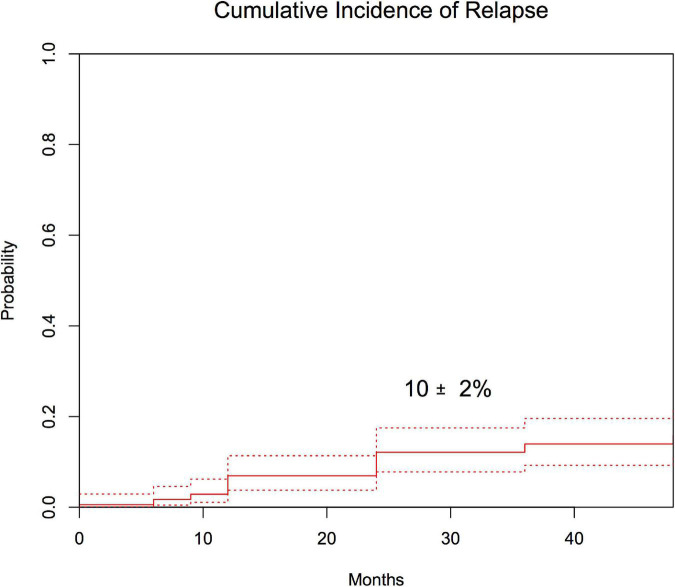
Cumulative incidence of CIR after 1 year post-transplantation.

**FIGURE 5:**
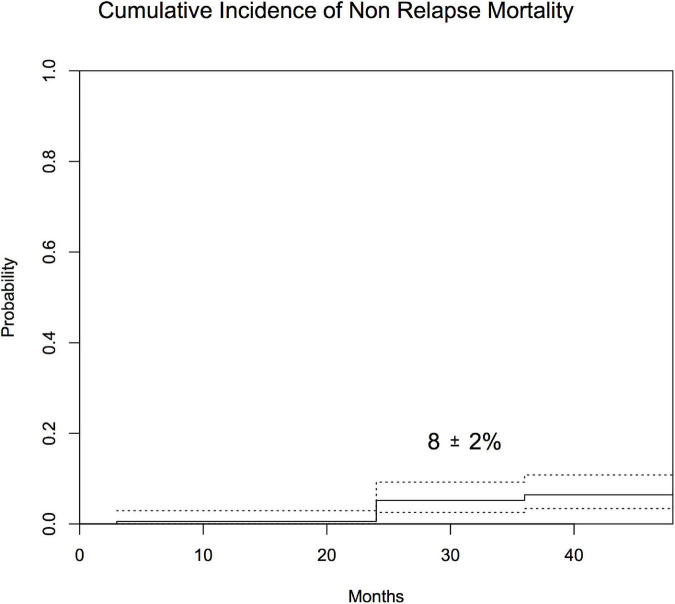
Cumulative incidence of NRM after 1 year post-transplantation.

Seventeen of the patients relapsed beyond 1 year after the transplantation. For the whole group, CIR was 10 ± 2% with a median time to relapse of 19 months (range: 14–54) (Figure 4). CIR was 12 ± 3% for patients with ALL, 9 ± 4% for those with AML, and 0% for those with CML. The cumulative incidence of NRM was 8 ± 2% with a median time to NRM of 18 months (range: 12–62) (Figure 5). NRM was 5 ± 3% for patients with AML, 9 ± 3% for those with ALL, and 14 ± 10% for those with CML. Variables that influenced NRM in the univariate analysis were as follows: aGvHD (yes vs. no, 17 ± 4% vs. 2 ± 1%; *p* = 0.001) and severe cGvHD (yes vs. no, 56 ± 13% vs. 4 ± 1%; *p* = 0.0001). When we analyzed immune reconstitution at 1 year after the transplant, we identified that some lymphocyte subsets were associated with NRM (Supplementary Figure 1). Patients with a B lymphocyte count below the median (421/μl) had a NRM of 15 ± 5% vs. 2 ± 2%; for those above median (*p* = 0.0005). Likewise, the amount of CD4+ and CD8+ subsets impacted on NRM; (T-cell count below median of 1,181/μl was associated with a NRM of 16 ± 5% vs. 2 ± 2% for those above median; *p* = 0.0029), (CD4+ cells below median of 483/μl had a NRM of 15 ± 5% vs. 2 ± 2% for those above median; *p* = 0.0001), (CD8+ below median 560/μl had 12 ± 5% of NRM vs. 6 ± 3% for those above the median; *p* = 0.018). A receiver operating characteristic curve for this risk factor is provided in Supplementary Figure 2. However, in the multivariate analysis, presence of severe cGvHD was associated with NRM (yes vs. no, HR 5; 95% CI, 1.92–13.16; *p* = 0.001).

The prognosis variables for relapse in the univariate analysis were disease phase (early phase 5 ± 2% vs. advanced phase 18 ± 4%; *p* = 0.018) and amount of CD4+ cells 2 years after transplantation (above the median of 837/μl 0% vs. 13 ± 5% for below the median; *p* = 0.02). We were unable to find any variable associated to relapse in the multivariate analysis.

Variables that influenced DFS in the univariate analysis were disease phase (early phase 87 ± 3% vs. advanced phase 74 ± 5%; *p* = 0.04), aGvHD (yes 73 ± 5% vs. no 87 ± 3%; *p* = 0.038), severe cGvHD (yes 41 ± 13% vs. no 85 ± 3%; *p* = 0.0001), and CD4+ lymphocytes at 2 years (above the median of 837/μl 98 ± 2% vs. below the median 82 ± 6%, *p* = 0.026). All patients who underwent an umbilical cord blood transplantation were alive and leukemia-free compared with those who underwent other types of transplant (UCB vs. other types 100% vs. 7 9 ± 3%, *p* = 0.079). However, in the multivariate analysis, the only variable that influenced DFS was presence of severe cGvHD (yes vs. no, HR 6.25; 95% CI, 1.35–34.48; *p* = 0.02). Patients who underwent a transplantation for ALL who had severe cGvHD and advanced-stage leukemia after the transplantation were related to worse DFS (HR, 5.44; 95% CI, 2.1–14.4; *p* = 0.001, and HR 0.36; 95% CI, 0.13–0.99; *p* = 0.049, respectively). However, for patients with AML, no variable influencing DFS was identified in the multivariate analysis.

## Discussion

Long-term follow-up studies provide clinicians relevant information to ensure surveillance and intervention for late complications and mortality in pediatric patients surviving an allogeneic transplant (11, 12). Landmark analysis on pediatric patients are especially important considering that a relevant proportion of them become lost to follow-up (13). Nonetheless, children and adults (4, 9) that survived beyond 2 years after transplant are at high risk of late mortality compared to the general population.

The main findings of this study are as follows. First of all, excellent OS and DFS were observed, higher than 80% for patients that survived 1 year after transplant with stable “plateau” during the study period. The good CRFS, which means that a high proportion of patients are alive and off therapy (14), is also remarkable. Despite this, a proportion of patients relapsed and/or died. As previously reported (1, 3, 4). Infections and relapse were the most common cause of late deaths in our series, and severe cGvHD was the third cause of death. It is noteworthy that there is no death due to second malignancies likely because most of the patients did not receive total body irradiation (15). Alternatively, longer follow-up may be needed for developing second malignancies after allogeneic transplant in this setting. We observed that the only variable that influenced DFS in the multivariate analysis was severe cGvHD mainly because it was associated with higher NRM and patients who received immunosuppressant treatment. cGvHD is a potentially modifiable factor by means of novel transplant approaches aimed to reduce risk factors for GvHD such as reduction in toxicity conditioning or T-cell graft manipulation among others. As we previously published (16), cGvHD is strongly associated with reduce risk of relapse, but only the classical mild and moderate chronic forms of GVHD have a positive impact on DFS (17).

Second, data on immune reconstitution shows that patients with a better immune reconstitution, especially on B and CD4+ T-cell population had a positive influence on DFS in univariate analysis mainly due to lower cumulative incidence of NRM. Better immune reconstitution in the early transplant period is associated with better transplant outcome. Jagasia et al. (18) showed that day + 30 absolute lymphocyte count was associated with improved survival, less relapse, less NRM, and less aGvHD in 160 children and adults with leukemia who received T-cell-depleted HSCs from an HLA-identical sibling donor. Socié et al. (16), in a univariate analysis, showed that high number of NK cells on day + 30 after haploidentical transplantation using CD3+/CD19+ depleted grafts in children reduced the rate of relapse. Several studies have shown that the quality and quantity of immune reconstitution are correlated with long-term clinical outcomes, including survival, presence of GvHD, viral diseases, and relapse rate, in both children and adults. Limited information is available about B cell reconstitution (19, 20) and its impact on transplant outcome. Our study strongly suggests that better immune reconstitution in the late period of transplant is associated with better outcome. However, only severe cGvHD had a definitive impact on DFS, which is not surprising because patients are at high risk of late mortality due to opportunistic infection while they are receiving immune suppressive treatment. Patients transplanted with ALL in advanced phase and with severe cGvHD are at the highest risk for late transplant failure resulting on lowest DFS.

Based on our findings, we cannot make any different recommendation for clinical surveillance compared with other previously published studies (18, 21). However, for patients with severe cGvHD, close clinical monitoring including immune reconstitution analysis, would be warranted to reduce late morbidity and mortality.

## Conclusion

The landmark analysis reveals that presence of severe cGvHD is the most important factor that negatively impacts on transplant outcomes in patients who survived beyond 1 year after the transplant. Transplant strategies should aim to reduce the risk of such a devastating long-term complication. Besides, immune reconstitution surveillance may help clinicians to better deal with late transplant complications.

## Data availability statement

The raw data supporting the conclusions of this article will be made available by the authors, without undue reservation.

## Ethics statement

Ethical review and approval was not required for the study on human participants in accordance with the local legislation and institutional requirements. Written informed consent to participate in this study was provided by the participants or their legal guardian/next of kin.

## Author contributions

BM, MG-V, and MD designed the study and analyzed the data, and wrote the manuscript. JR, AP, IL, and MR designed the study, provide and revised the data, and contributed to the data analysis. All authors approved final manuscript version.

## Supplementary material

Supplementary Figure 1 Immune reconstitution and non-relapse mortality. **(A)** One-year T lymphocytes/μl. **(B)** One-year CD8 lymphocytes/μ. **(C)** One-year CD4 lymphocytes/μl. **(D)** One-year B lymphocytes/μl. Yes means patient died because of non-relapse causes.

Supplementary Figure 2 Receiver operating characteristic(ROC) curve. The 80% specificity for CD4+ cells is 786/μl, and the area under the ROC curve (AUC) is 0.74 (0.6–0.88).

## References

[1] Cho C Hsu M Barba P Maloy MA Avecilla ST Barker JN Long-term prognosis for 1-year relapse-free survivors of CD34+ cell-selected allogeneic hematopoietic stem cell transplantation: a landmark analysis. *Bone Marrow Transplant.* (2017) 52:1629–36. 10.1038/bmt.2017.197 28991247 PMC5718946

[2] Wingard JR Majhail NS Brazauskas R Wang Z Sobocinski KA Jacobsohn D Long-term survival and late deaths after allogeneic hematopoietic cell transplantation. *J ClinOncol.* (2011) 29:2230–9. 10.1200/JCO.2010.33.7212 PMC3107742 21464398

[3] Bitan M Ahn KW Millard HR Pulsipher MA Abdel-Azim H Auletta JJ Personalized prognostic factors risk score for long-term survival for children with acute leukemia after allogeneic transplant. *Biol Blood Marrow Transplant.* (2017) 23:1523–30. 10.1016/j.bbmt.2017.05.011 28527984 PMC5683075

[4] Holmqvist AS Chen Y Wu J Kung M Ness E Parman M Late mortality after allogeneic blood or marrow transplantation in childhood for leukemia: a report from the blood or marrow transplant survivor study-2. *Leukemia.* (2018) 32:2706–9. 10.1038/s41375-018-0171-4 29946190 PMC9990491

[5] Gonzalez-Vicent M Molina B Andión M Sevilla J Ramirez M Pérez A Allogeneic hematopoietic transplantation using haploidentical donor vs. unrelated cord blood donor in pediatric patients: a single-center retrospective study. *Eur J Haematol.* (2011) 87:46–53. 10.1111/j.1600-0609.2011.01627.x 21692851

[6] Díaz MA Lopez I Molina B Pereto A Zubicaray J Sevilla J Graft failure after ‘ex-vivo’ T-cell depleted haploidentical transplantation in pediatric patients with high-risk hematological malignancies. A risk factors and outcomes analysis. *Leuk Lymphoma.* (2021) 62:3130–7. 10.1080/10428194.2021.196.3018 34263704

[7] Diaz MA Pérez-Martínez A Herrero B Deltoro N Martinez I Ramirez M Prognostic factors and outcomes for pediatric patients receiving an haploidentical relative allogeneic transplant using CD3/CD19-depleted grafts. *Bone Marrow Transplant.* (2016) 51:1211–6. 10.1038/bmt.2016.101 27088380

[8] Diaz M Gonzalez-Vicent M Gonzalez M Verdeguer A Martinez A Perez-Hurtado M Long-term outcome of allogeneic PBSC transplantation in pediatric patients with hematological malignancies: a report of the Spanish working party for blood and marrow transplantation in children (GETMON) and the Spanish group for allogeneic peripheral blood transplantation (GETH). *Bone Marrow Transplant.* (2005) 36:781–5. 10.1038/sj.bmt.1705135 16151427

[9] Pérez-Martínez A González-Vicent M Valentín J Aleo E Lassaletta A Sevilla J Early evaluation of immune reconstitutionfollowing allogeneic CD3/CD19-depleted grafts from alternativedonors in childhood acute leukemia. *Bone Marrow Transplant.* (2012) 47:1419–27. 10.1038/bmt.2012.43 22410752

[10] Przepiorka D Weisdorf D Martin P Klingemann HG Beatty P Hows J 1994 consensus conference on acute GVHD grading. *Bone Marrow Transplant.* (1995) 15:825–8. 7581076

[11] Baker KS Bresters D Sande JE. The burden of cure: long-term side effects following hematopoieticstem cell transplantation (HSCT) in children. *PediatrClin North Am.* (2010) 57:323–42. 10.1016/j.pcl.2009.11.008 20307723

[12] Jacobsohn DA Arora M Klein JP Hassebroek A Flowers ME Cutler CS Risk factors associated with increased nonrelapse mortality andwith poor overall survival in children with chronic graft-versus-host disease. *Blood.* (2011) 118:4472–9. 10.1182/blood-2011-04-349068 21878671 PMC3204914

[13] Buchbinder D Brazauskas R Bo-Subait K Ballen K Parsons S John T Pedrictors of loss of follow-up among pediatric and adult hematopoietic cell transplantation survivors: a report from the center for international blood and marrow transplant research. *Biol Blood Marrow Transplant.* (2020) 26:553–61. 10.1016/j.bbmt.2019.11.003 31726205 PMC7367505

[14] Mehta RS Holtan SG Wang T Hemmer MT Spellman SR Arora M Composite GRFS and CRFS outcomes after adult alternative donor HCT. *J ClinOncol.* (2020) 38:2062–76. 10.1200/JCO.19.00396 32364845 PMC7302955

[15] Baker KS Leisenring WM Goodman PJ Ermoian RP Flowers ME Schoch G Total body irradiation dose and risk of subsequentneoplasms following allogeneic hematopoietic cell transplantation. *Blood.* (2019) 133:2790–9. 10.1182/blood.2018874115 30992266 PMC6598379

[16] Socié G Stone JV Wingard JR Weisdorf D Henslee-Downey PJ Bredeson C Long-term survival and late deaths after allogeneic bone marrow transplantation: late effects working committee of the International Bone Marrow Transplant Registry. *N Engl J Med.* (1999) 341:14–21. 10.1056/NEJM199907013410103 10387937

[17] Abdel-Azim H Elshoury A Mahadeo KM Parkman R Kapoor N. Humoral immune reconstitution kinetics after allogeneic hematopoietic stem cell transplantation in children: a maturation block of IgM Memory B cells may lead to impaired antibody immune reconstitution. *Biol Blood Marrow Transplant.* (2017) 23:1437–46. 10.1016/j.bbmt.2017.05.005 28495643

[18] Jagasia MH Greinix HT Arora M Williams KM Wolff D Cowen EW National institutes of health consensus development project on criteria for clinical trials in chronic graft-versus-host disease: I. The 2014 diagnosis and staging working group report. *Biol Blood Marrow Transplant.* (2015) 21:389–401. 10.1016/j.bbmt.2014.12.001 25529383 PMC4329079

[19] Molina B Gonzalez-Vicent M Herrero B Deltoro N Ruiz J Perez Martinez A Kinetics and risk factors of relapse after allogeneic stem cell transplantation in children with leukemia: a long-term follow-up single-center study. *Biol Bone Marrow Transplant.* (2019) 25:100–6. 10.1016/j.bbmt.2018.08.012 30142415

[20] Savani BN Mielke S Rezvani K Montero A Yong AS Wish L Absolute lymphocyte count on day 30 is a surrogate for robust hematopoietic recovery and strongly predicts outcome after T cell-depleted allogeneic stem cell transplantation. *BiolBlood MarrowTransplant.* (2007) 13:1216–23. 10.1016/j.bbmt.2007.07.005 PMC3426353 17889359

[21] Hashmi SK Lee SJ Savani BN Burns L Wingard JR Perales MA ASBMT practice guidelines committee survey on long-termfollow-up clinics for hematopoietic cell transplant survivors. *Biol Blood Marrow Transplant.* (2018) 24:1119–24. 10.1016/j.bbmt.2018.03.023 29608957

