# Supplementary figures and images for: Long-term transplant outcomes after allogeneic hematopoietic transplant in pediatric patients with hematological malignancies are influenced by severe chronic graft vs. host disease and immune reconstitution
Source: Front Pediatr. 2022 Aug 12;10:947531. doi: 10.3389/fped.2022.947531 (PMC9411718; doi:10.3389/fped.2022.947531)

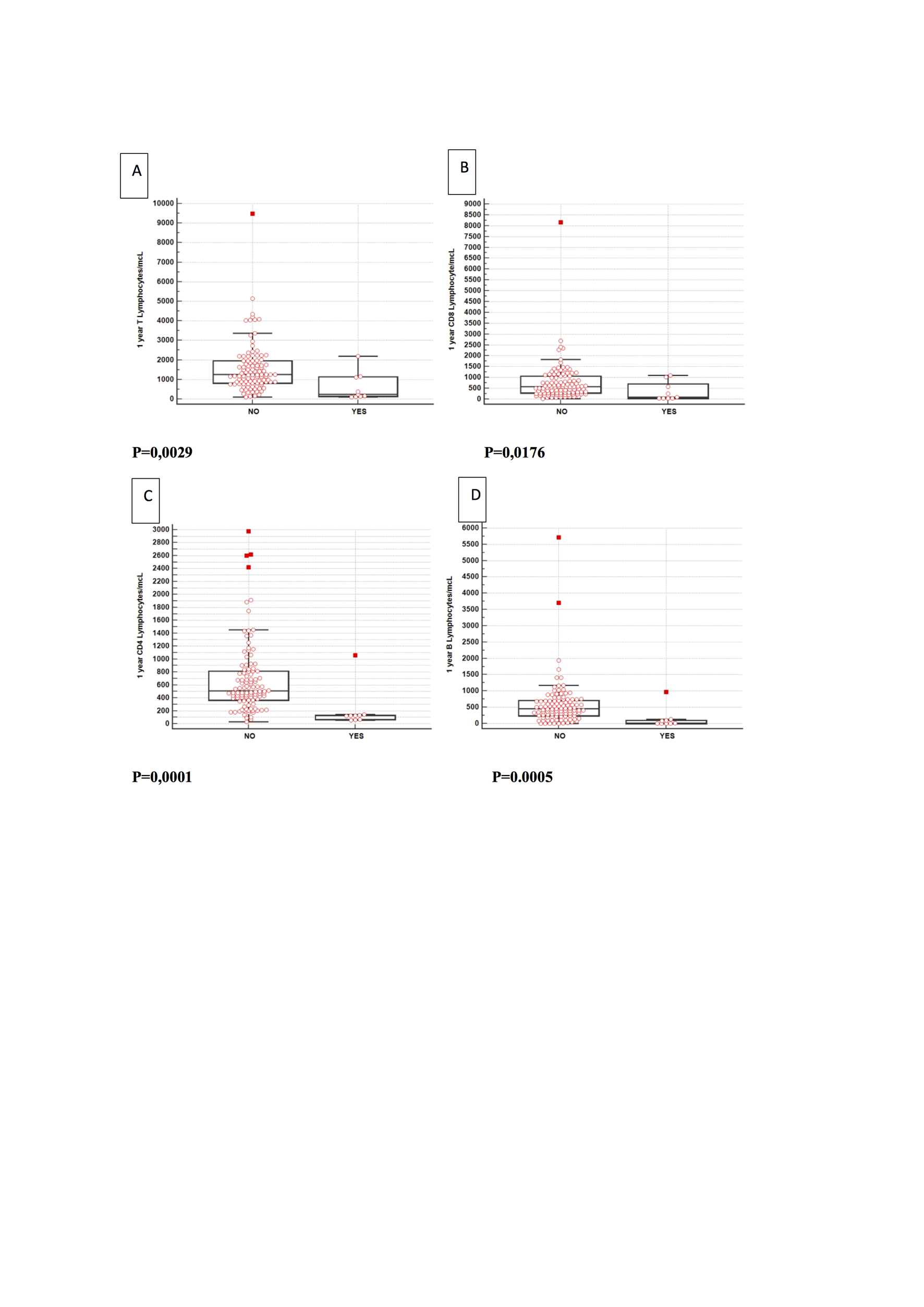

Supplement: Supplementary Figure 1 — Immune reconstitution and non-relapse mortality. (A) One-year T lymphocytes/μl. (B) One-year CD8 lymphocytes/μ. (C) One-year CD4 lymphocytes/μl. (D) One-year B lymphocytes/μl. Yes means patient died because of non-relapse causes. [file Image_1.TIFF]

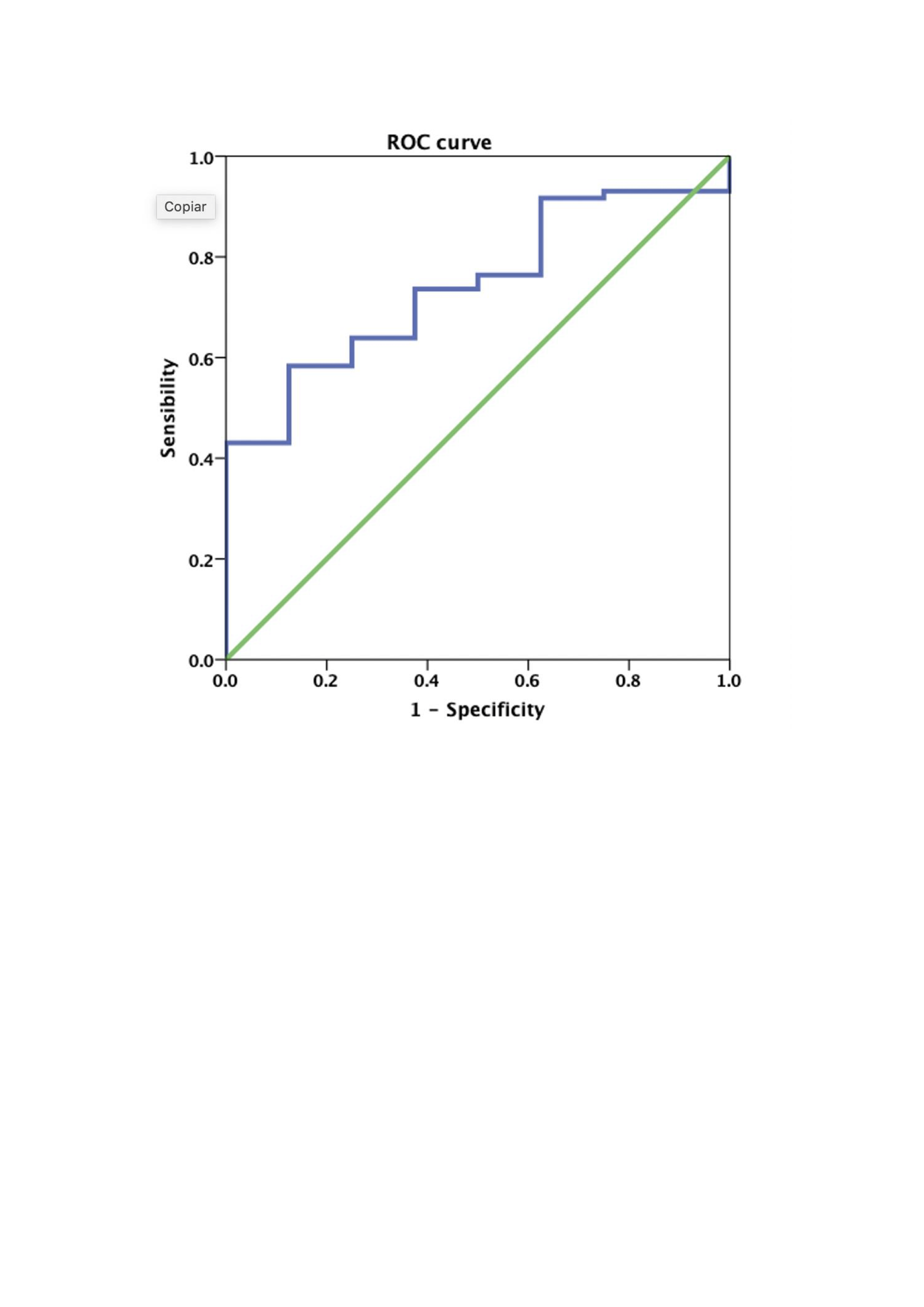

Supplement: Supplementary Figure 2 — Receiver operating characteristic(ROC) curve. The 80% specificity for CD4+ cells is 786/μl, and the area under the ROC curve (AUC) is 0.74 (0.6–0.88). [file Image_2.TIFF]
